# Protective effect of porphyra-334 on UVA-induced photoaging in human skin fibroblasts

**DOI:** 10.3892/ijmm.2014.1815

**Published:** 2014-06-19

**Authors:** JINA RYU, SU-JIN PARK, IN-HYE KIM, YOUN HEE CHOI, TAEK-JEONG NAM

**Affiliations:** 1 Department of Food and Life Science, Pukyong National University, Busan 608-737, Republic of Korea; 2 Institute of Fisheries Sciences, Pukyong National University, Busan 619-911, Republic of Korea

**Keywords:** porphyra-334, UVA, anti-photoaging, human skin fibroblasts

## Abstract

The significant increase in life expectancy is closely related to the growing interest in the impact of aging on the function and appearance of the skin. Skin aging is influenced by several factors, and solar ultraviolet (UV) irradiation is considered one of the most important causes of skin photoaging. The aim of this study was to examine the anti-photoaging role of porphyra-334 from *Porphyra (P.) yezoensis*, a mycosporine-like amino acid (MAA), using high-performance liquid chromatography (HPLC), and electrospray ionization-mass spectrometry (ESI-MS). In the present study, extracted UV-absorbing compounds from *P. yezoensis* included palythine, asterina-330 and porphyra-334. Porphyra-334 was the most abundant MAA in *P. yezoensis*, and it was therefore used for conducting antiphotoaging experiments. The effect of porphyra-334 on the prevention of photoaging was investigated by measuring reactive oxygen species (ROS) production and matrix metalloproteinase (MMP) levels, as well as extracellular matrix (ECM) components and protein expression in UVA-irradiated human skin fibroblasts. Porphyra-334 suppressed ROS production and the expression of MMPs following UVA irradiation, while increasing levels of ECM components, such as procollagen, type I collagen, elastin. These results suggest that porphyra-334 has various applications in cosmetics and toiletries because of its anti-photoaging activities and may serve as a novel anti-aging agent.

## Introduction

Intrinsic and extrinsic aging are two basic processes of skin aging. Extrinsic aging is generally referred to as photoaging and is characterized by severe wrinkling and pigmentary changes, such as solar lentigo and mottled pigmentation on exposed areas, such as the face, neck and forearms. Solar ultraviolet (UV) irradiation is a major environmental hazard that generates reactive oxygen species (ROS), induces DNA damage, and ultimately results in skin inflammation, photoaging, and cancer development (1).

Oxidative stress is considered a primary feature in aging and age-related diseases, including cataracts, atherosclerosis, diabetes and Alzheimer’s disease. Aging is considered to be the consequence of free radical damage by various endogenous ROS, according to the original free radical theory of aging. ROS production and release can be affected by environmental factors such as UV radiation and exogenous toxins. Cytoprotective responses are characterized by the upregulation of antioxidant enzymes and decreased sensitivity to oxidative stress damage (2). Various compounds with differential antioxidant properties are found in plants, which may be applicable as therapeutics to decrease and prevent free radical damage. Several medical plants have been screened and assessed for properties in antagonizing free radical-induced oxidative stress, and their natural products are used to treat 87% of all classified human diseases (3,4).

Recently, it was suggested that excessive matrix degradation by UV-induced matrix metalloproteinases (MMPs), which are secreted by various cells including keratinocytes, fibroblasts and inflammatory cells, contributes to connective tissue damage during photoaging (5–8). MMPs are a family of enzymes responsible for degrading connective tissue. They are structurally related endopeptidases that mediate the degradation of different macromolecular components of the extracellular matrix (ECM) and the basement membrane, including collagen. The UV-induced synthesis of MMPs present on dermal fibroblasts contributes to the breakdown of dermal interstitial collagen and other connective tissue components. These results are indicative of the MMP-mediated degradation of collagen in photodamaged skin. In particular, in photodamaged aging skin, increased ROS leads to the induction of AP-1 and NF-κB transcription factors, which consequently induce collagen degradation by the upregulation of MMPs. These properties make MMPs an attractive target for anti-photoaging compounds. ECM degrades naturally over time due to intrinsic aging. However, this breakdown is accelerated by external factors (especially UV irradiation) and the resultant oxidative stress, as well as increases in activity of MMPs.

Fibroblasts are the major cell component of the dermis that produces ECM proteins, as well as structural proteins (collagen and elastin), adhesive proteins (laminins and fibronectin), glycosaminoglycans (GAG) and proteoglycans. The most abundant structural protein in skin connective tissue is type I collagen (90% of ECM in dermis), which is synthesized primarily by fibroblasts and is responsible for conferring strength and resilience to cells (9). Elastin is an essential part of various human tissues that depend on elasticity, including the skin, lung and arteries. Elastin provides these elastic tissues with the ability to stretch and recoil, and it plays a critical role in supporting and maintaining healthy cells (10).

Mycosporine-like amino acids (MAAs) possess significant chemoprotective effects against photo-induced skin senescence (11). MAAs found in and isolated from a number of marine organisms, such as cyanobacteria, algae, and heterotrophic bacteria, have attracted a great deal of interest, especially for potential UV protection. In a recent study, it was suggested that MAAs have antioxidant properties and UV absorbance activity (12). An important MAA is porphyra-334, which has been reported to act mainly in photoprotection, but it also posseses antioxidation abilities. Results of a recent study have shown that algae extracts prevent UV-induced photodamage in human keratinocytes (13). Although the photo-protective effect of MAAs in algae and tissue has been reported (14,15), little is known regarding its effects on the aging process of skin cells. This study investigated whether supplementation with porphyra-334, an active MAA from *Porphyra (P.) yezoensis*, inhibits UVA-induced cellular senescence in human skin fibroblasts.

## Materials and methods

### Extraction and isolation of water-soluble porphyra-334

The porphyra-334 extraction method was performed as previously described (16) with minor modifications. Briefly, dried *P. yezoensis* (100 g) was ground and extracted in hydrophilic solvent consisting of 80% aqueous methanol (v/v) at 45°C for 2 h. The extract was filtered (no. 3, 90 mm; Advantec, Tokyo, Japan) to remove powder particles, and the residual aqueous suspension was evaporated to dryness under vacuum at 41°C (EYELA N-1100; Tokyo Rikakikai Co., Ltd., Nihonbashi Honcho, Japan). The dried extract was dissolved in 150 ml ultrapure water and transferred to a separating funnel containing 666 ml chloroform-methanol-ultrapure water (2:1:1, v/v/v). The upper layer containing crude MAAs was collected. The water layer was then filtered through 0.2-μm pore-sized syringe filters (Woongki Science, Seoul, Korea) and loaded onto a Strata C18-E cartridge (Phenomenex, Inc., Torrance, CA, USA) (previously equilibrated with ultrapure water) for analysis.

### High-performance liquid chromatography (HPLC) analysis

Porphyra-334 was purified using an Agilent 1100 series HPLC system equipped with a diode array detector (DAD; Agilent Technologies, Inc., Palo Alto, CA, USA). The HPLC conditions used were: column, Gemini-NX 5 μ C18 (i.d. 250x21.20 mm; Phenomenex); column temperature, RT; flow rate, 30 ml/min; mobile phase, 0.1% acetic acid in H_2_O; and wavelength for detection, 334 nm. Purified porphyra-334 was stored in the dark at −70°C until analysis. Identification of MAAs was performed using UV absorption spectra and mass spectrometry.

### Electrospray ionization-mass spectrometry (ESI-MS) analysis

To identify the peaks of fingerprints, the Agilent 1100 series (Agilent Technologies) ion-trap mass spectrometer with an electrospray ionization (ESI) source was used for the HPLC/MS method. ESI-MS conditions of each HPLC peak were set as follows: scan mass range, *m/z* 100–600; fragmentor voltage, 70 V; drying gas N_2_ flow rate, 12 l/min; sheath gas flow rate, 60 arbitrary units; drying gas temperature, 350°C; and capillary voltage, 3,000 V.

### Cell culture

Human skin fibroblasts (CCD-986sk) were obtained from the American Type Culture Collection (ATCC, Manassas, VA, USA). Cells were grown in Dulbecco’s modified Eagle’s medium (DMEM; Gibco, Grand Island, NY, USA) containing 10% (v/v) fetal bovine serum (FBS; HyClone, Logan, UT, USA) and 1% (v/v) penicillin-streptomycin (Gibco) under a humidified atmosphere of 5% CO_2_ at 37°C.

### UVA irradiation and treatment

Prior to UV irradiation, cells were washed with PBS and exposed to a radiation dose of 10 J/cm^2^ of UVA light (BLX-254; Vilber Lourmat, Marne La Vallée, France) in PBS. Subsequent to irradiation, the treated cells were washed with PBS and replaced with different concentrations of porphyra-334 for 24 h. Concomitantly, no irradiation control cells were treated in the same manner, although the wells were covered with aluminum foil to prevent irradiation.

### Cell viability

Cell viability was determined using a 3-(4,5-dimethylthiazol-2-yl)-5-(3-carboxymethoxy-phenyl)-2- (4-sulfophenyl)-2H-tetrazolium (MTS) assay (Promega Corp., Madison, WI, USA) according to the manufacturer’s instructions. Briefly, 2x10^4^ cells/well were plated and allowed to attach to 96-well plates. The cells were then exposed to serial concentrations of porphyra-334 for 24 h. After 24 h, a soluble MTS reagent was added and absorbance of the formazan was measured directly in 96-well plates at 490 nm using a multi-plate reader (SpectraMAX 340PC; Molecular Devices, Sunnyvale, CA, USA). Relative cell viability was calculated as the percent viability relative to the untreated control cells. Each experiment was performed in triplicate.

### Senescence-associated β-galactosidase (SA-β-gal) staining

SA-β-gal activity was determined at 24 h after UVA irradiation. A cellular senescence assay kit (Cell Biolabs, Inc., San Diego, CA, USA) was performed according to the manufacturer’s instructions. Briefly, the cells were washed twice in PBS and incubated at room temperature for 5 min with fixing solution. The cells were washed three times with PBS, the final wash was aspirated, and the cells were completely covered with freshly prepared cell staining working solution. The cells were then incubated in the dark overnight at 37°C. Following removal of the cell staining solution, the cells were washed twice with PBS and blue-stained senescence cells were observed using a light microscope (Olympus Microscope System IX51; Olympus, Tokyo, Japan).

### Intracellular ROS production

The production of intracellular ROS was measured using the redox-sensitive fluorescent dye 2′-7′-dichlorofluorescein diacetate [DCF-DA (C_24_H_14_C_l2_O_7_); Sigma-Aldrich, Inc., St. Louis, MO, USA]. The ability of cells to produce ROS was measured by fluorescence. The cells were treated with 25 μM DCF-DA for 30 min at 37°C and washed twice in PBS. Representative images were obtained using a fluorescence microscope (Olympus Microscope System IX51; Olympus).

### Elastase activity

Elastase activity using the synthetic substrate N-Succinyl-Ala-Ala-Ala-p-nitroanilide (STANA; Sigma-Aldrich) was measured as previously described (17). Briefly, 100 μl of enzyme solution was dispensed into 96-well plates, which were pre-incubated for 15 min at 37°C. Following the addition of 2 μl 55.3 mM STANA, the plates were further incubated for 1 h at 37°C. The release of p-nitroaniline was measured by absorbance at 410 nm and enzymatic activity was expressed as a percentage of total p-nitroaniline.

### Total collagen

Total collagen synthesis in fibroblasts was measured using the Procollagen Type I C-Peptide (PIP) EIA kit (Takara Bio Inc., Otsu, Japan) according to the manufacturer’s instructions. Briefly, 100 μl of antibody-POD conjugate solution were added to appropriate wells, after which 20 μl of cell culture medium was added to the wells within 5 min and incubated for 3 h at 37°C. The contents were removed by suction and the wells were washed four times with 300 μl of washing buffer. Substrate solution (100 μl) was added to each well and incubated at room temperature for 15 min. Subsequently, 100 μl of stop solution was added to all the wells, and absorbance was read at 450 nm.

### Reverse transcription-polymerase chain reaction (RT-PCR)

Total RNA from each sample was extracted using TRIzol reagent (Invitrogen, Carlsbad, CA, USA). According to the manufacturer’s instructions, total RNA (1 μg) was subjected to first strand cDNA synthesis using a Reverse Transcriptase PreMix kit (Intron Biotechnology, Inc., Gyeonggi-do, Korea). PCR amplification of the cDNA products was performed with 2X TOPsimple™ DyeMIX(aliquot)-*n*Taq (Enzynomics, Daejeon, Korea) and primer pairs. Amplified products were separated by 1% agarose gel electrophoresis and visualized with 1 mg/ml ethidium bromide. mRNA levels were normalized using GAPDH as an internal control.

### Western blot analysis

After treatment, cells were washed twice with PBS, harvested and lysed in RIPA buffer [50 mM Tris (pH 7.4), 1 mM ethylene glycol tetraacetic acid (EGTA), 150 mM NaCl, 1% Triton X-100, 0.25% sodium deoxycholate]containing protease inhibitor cocktail (Geno Technology, Inc., St. Louis, MO, USA). The lysates were centrifuged at 13,475 x g for 15 min at 4°C (Smart-R17; Hanil Science Industrial, Incheon, Korea). Supernatants were collected and their protein concentrations were determined using a BCA protein assay kit (Pierce Biotechnology, Inc., Rockford, IL, USA). Equal amounts of protein (30 μg) were boiled for 10 min and separated using 7.5–15% sodium dodecyl sulfate-polyacrylamide gel electrophoresis (SDS-PAGE). The resolved proteins were then transferred to polyvinylidene difluoride (PVDF) membranes (Millipore Corp., Billerica, MA, USA). The membranes were blocked by incubation with 1% bovine serum albumin (BSA) in TBS-T [10 mM Tris-HCl, 150 mM NaCl (pH 7.5) containing 0.1% Tween-20] at room temperature for 1 h and incubated with specific primary antibody (Santa Cruz Biotechnology, Inc., Santa Cruz, CA, USA) for 3 h. The membranes were washed three times with TBS-T and incubated for 2 h with the appropriate HRP-conjugated goat anti-rabbit, goat anti-mouse or rabbit anti-goat secondary antibody (Santa Cruz Biotechnology) diluted at 1:10,000 in TBS-T containing 1% BSA. The respective proteins were detected with SuperSignal^®^ West Pico (Thermo Fisher Scientific, Inc., Rockford, IL, USA). Equal protein loading was assessed by the detection of GAPDH levels.

### Statistical analysis

The results were presented as means ± SEM from at least three independent experiments. Data were analyzed using one-way analysis of variance (ANOVA) with the Student’s t-test using SPSS 10.0 (SPSS, Inc., Chicago, IL, USA). Differences were considered significant at p<0.05.

## Results

### Identification of MAAs

**Figure 1:**
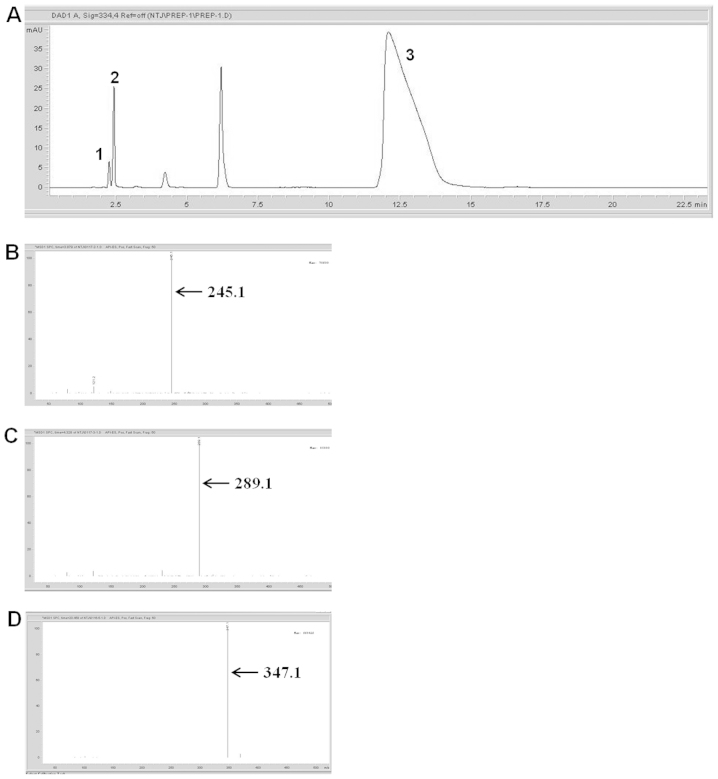
Chromatographic separation of mycosporine-like amino acids (MAAs) from *Porphyra (P.) yezoensis*. (A) High-performance liquid chromatography (HPLC) chromatogram of *P. yezoensis*, showing the peaks for peak 1 (2.29 min), peak 2 (2.493 min) and peak 3 (11.53 min). Mass spectrometry (MS) spectra and chemical structures of peaks 1, 2 and 3 exhibits [M+H]^+^ ions at *m/z* (B) 245.1, (C) 289.1 and (D) 347.1.

As shown in Fig. 1A, the HPLC analysis of *P. yezoensis* revealed various absorption peaks at 334 nm. Peaks were identified tentatively by comparing retention times with known standards (peak 1, 2.29 min; peak 2, 2.493 min; and peak 3, 11.53 min). We then identified the three peaks using LC/MS analysis. Peak 1, 2 and 3 showed [M+H]^+^ ions at *m/z* 245.1, 289.1 and 347.1. Peak 1 was tentatively identified as palythine, peak 2 as asterina-330 and peak 3 as porphyra-334. Porphyra-334 was the most abundant MAA in *P. yezoensis*. Therefore, the following photoaging protection experiments were conducted using porphyra-334.

### Effect of porphyra-334 on cell viability

**Figure 2:**
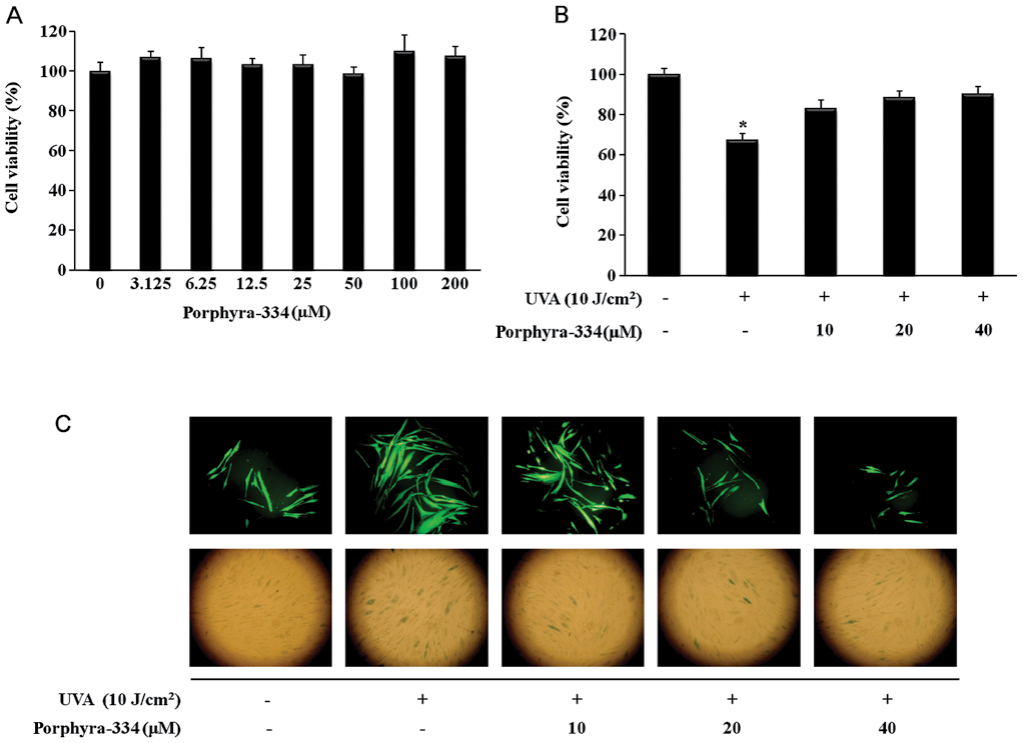
Effect of porphyra-334 on UVA-induced cell viability, reactive oxygen species (ROS) generation and senescence-associated β-galactosidase (SA-β-gal) expression in human skin fibroblasts. (A) Porphyra-334 (0–200 μM) did not exert cytotoxic effects on the proliferation of fibroblasts. (B) Cells were exposed to UVA (10 J/cm^2^) irradiation, and cell viability (%) was determined. (C) Porphyra-334 treatment inhibited the production of intracellular ROS and the activity of SA-β-gal in a dose-dependent manner. ^*^P<0.05 compared with the only non-UVA irradiated group.

To determine the cytotoxic effect of porphyra-334 on human skin fibroblasts, cell viability was measured using the MTS assay. Human skin fibroblasts were incubated with or without porphyra-334 at concentrations of 0–200 μM. As shown in Fig. 2A, no significant toxicity was observed in the cells treated with porphyra-334 for 24 h. To explore the protective effect of porphyra-334 on UVA-induced cell damage, human skin fibroblasts (previously exposed to UVA irradiation) were incubated with various concentrations of porphyra-334 (0–40 μM). As shown in Fig. 2B, treatment with porphyra-334 protected against cell damage in a dose-dependent manner.

### Effect of porphyra-334 on ROS generation

To determine whether porphyra-334 functions as a scavenger of UVA-induced ROS generation, intracellular ROS levels were measured. As shown in Fig. 2C (upper panel), the level of ROS in UVA-irradiated cells increased compared with non-irradiated cells. UVA-exposed cells showed a significant reduction in DCF-DA staining in the presence of porphyra-334, indicating that porphyra-334 inhibited the intracellular accumulation of ROS in human skin fibroblasts damaged by UVA-induced oxidant stress.

### Effect of porphyra-334 on SA-β-gal activity

SA-β-gal staining was performed to observe SA-β-gal activity, which is a biomarker of senescence. Images clearly indicated that the cells were induced to a senescence-like state by UVA irradiation. The inhibitory activity of porphyra-334 was also observed and found to effectively suppress the expression of SA-β-gal in a dose-dependent manner (Fig. 2C lower panel).

### Effect of porphyra-334 on MMP expression

**Figure 3:**
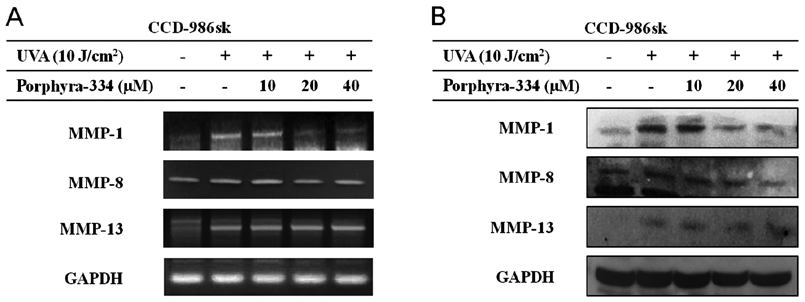
Effect of porphyra-334 on UVA-induced production of matrix metalloproteinases (MMPs) in human skin fibroblasts. Following UVA irradiation at 10 J/cm^2^, cells were treated with 10, 20 and 40 μM porphyra-334 for 24 h, and MMP expression levels were measured by reverse transcription-polymerase chain reaction (RT-PCR) and western blot analysis. (A) RT-PCR results revealed an association between porphyra-334 concentrations and MMP mRNA expression. mRNA expression was normalized to the housekeeping gene, GAPDH. (B) Western blot analysis revealed that porphyra-334 prevented the UVA-induced expression of MMPs. GAPDH was the loading control for western blot analysis.

To determine whether porphyra-334 inhibited MMP expression induced by UVA irradiation, human skin fibroblasts were irradiated with UVA (10 J/cm^2^) and treated with porphyra-334 for 24 h. Based on RT-PCR, UVA irradiation significantly increased MMP-1 mRNA expression in culture medium (Fig. 3A). To investigate the dose-dependent effect of porphyra-334, the cells were treated with different concentrations of porphyra-334 ranging from 10 to 40 μM. The highest concentration of porphyra-334 inhibited MMP-1 mRNA expression in UVA irradiated human skin fibroblasts up to 56.2%, and the inhibition was dose-dependent. The inhibition of MMP-8 was similar to MMP-1, but not MMP-13. MMP-13 expression in the presence of porphyra-334 was similar to the UVA-irradiation control. Porphyra-334 reduced the elevated MMP expression, with the exception of MMP-13, at the gene and protein levels compared with the control groups, which were irradiated without treatment. Of the three MMPs, MMP-1 was the most active compared with the positive control. Thus, porphyra-334 exerted a protective effect on UVA-induced collagen degradation via the negative regulation of MMP expression.

### Effect of porphyra-334 on UVA-induced intracellular procollagen

**Figure 4:**
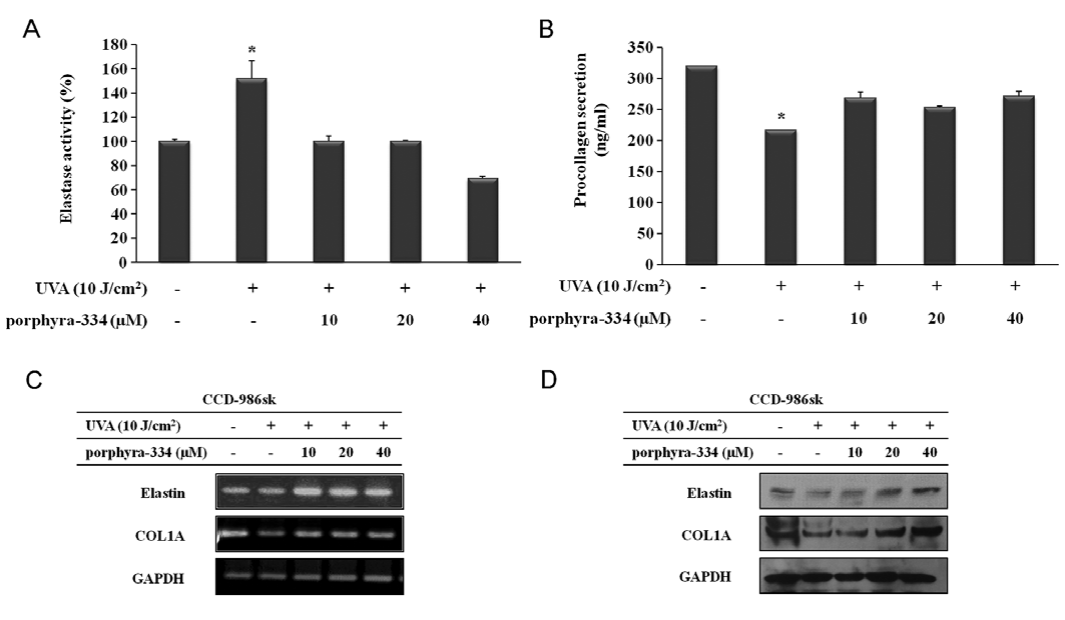
Effect of porphyra-334 on the UVA-induced degradation of elastin and collagen in human skin fibroblasts. Following UVA irradiation at 10 J/cm^2^, the cells were treated with 10, 20 and 40 μM of porphyra-334 for 24 h. (A) The procollagen levels and (B) elastase activity was measured by ELISA. (C) The mRNA transcription and (D) protein level of type I collagen and elastin expression levels were measured by reverse transcription-polymerase chain reaction (RT-PCR) and western blot analysis. ^*^P<0.05 compared with the only non-UVA irradiated group.

To evaluate the effect of porphyra-334 on collagen synthesis, human skin fibroblasts were treated with various concentrations of porphyra-334 (0–40 μM) for >24 h. The secreted procollagen level was measured in the culture medium using an ELISA assay, as described in the ‘Materials and methods’. As shown in Fig. 4A, the collagen content was 319.5±0.069 ng/ml in the non-irradiated cells but 217.333±0.177 ng/ml in the UVA-irradiated cells. Procollagen secretion levels increased by 269.167±9.090, 253.833±1.464 and 271.833±7.224 ng/ml in the presence of porphyra-334 at concentrations of 10, 20 and 40 μM, respectively. Thus, porphyra-334 has a protective effect on collagen degradation by enhancing collagen synthesis in photodamaged human skin fibroblasts.

### Effect of porphyra-334 on UVA-induced elastase activity

UV irradiation is known to cause elastin degradation by activating elastase (18), and loss of skin elastin caused by UVA irradiation results in wrinkle formation. In this study, the elastase activity of human skin fibroblasts in response to porphyra-334 was confirmed based on the release of p-nitroaniline. As shown in Fig. 4B, elastase activity increased by 51.9% in the supernatant and intracellular contents, respectively, following UVA irradiation compared with the non-irradiated cells. This increase in the elastase inhibitory effect was decreased by porphyra-334 treatment at 10, 20 and 40 μM after UVA irradiation by 51.9, 51.9 and 82.5% compared with the UVA-irradiated cells, respectively.

### Effect of porphyra-334 on UVA-induced collagen and elastin degradation

To examine the effect of porphyra-334 on collagen and elastin degradation, human skin fibroblasts previously stimulated with UVA irradiation were incubated with various concentrations of porphyra-334 (0–40 μM). The expression of the specific elastin and type I collagen at the mRNA and protein levels was determined by RT-PCR and western blot analysis, respectively. The mRNA and protein levels of type I collagen and elastin are shown in Fig. 4C and D. Expression of type I collagen was decreased in UVA-irradiated cells, while the decrease in cellular collagen levels following UVA exposure was prevented in a dose-dependent manner in the presence of porphyra-334. Similarly, the expression of elastin was enhanced following porphyra-334 treatment after UVA irradiation. Results of the western blot analysis were in concordance with those of RT-PCR. These results indicated that porphyra-334 may be involved in collagen synthesis by regulating collagen-degrading MMP expression and elastinase activity.

## Discussion

*Porphyra sp.* has been used to protect against a variety of diseases. However, the intracellular signaling and protective effects of porphyra-334 against UVA-induced photodamage in human skin fibroblasts remains poorly understood. Numerous studies (19–21) have explored the impact of UV irradiation on skin cells, but the beneficial effect of photoprotective agents on UVA-induced damage in human skin is not well characterized. UVA is a potent inducer of various ROS, and it causes lipid peroxidation in cell membranes. Antioxidant defense mechanisms may be overwhelmed by excessive free radical generation, which damages the cells and increases the chances of photocarcinogenesis. Development of novel antioxidant strategies to supplement the natural defense mechanism of the skin may be an important strategy to reduce UV-induced effects. This study showed that porphyra-334, a rich source of MAA derived from *P. yezoensis*, is capable of reducing the adverse effects of UVA-mediated cutaneous damage.

Photoaging associated with UV irradiation is thought to play a central role in initiating and driving the signaling events that lead to cell responses. UV radiation of skin decreases antioxidant enzyme concentrations (22) and increases hydrogen peroxides and other ROS (23,24). UV irradiation initiates the generation of ROS and alters gene and protein structure and function, leading to skin damage. Porphyra-334 was shown to dose-dependently decrease intracellular UVA-induced ROS generation in human skin fibroblasts based on a modified DCF-DA fluorescence assay. Since the generation of MMPs is significantly induced by ROS (25), we showed that the ROS scavenging of porphyra-334 was comparable to the non-UV irradiated control. This suggests that porphyra-334 controls the expression of MMPs by scavenging excess ROS in damaged skin fibroblasts.

UV irradiation enhances the decomposition of skin connective tissue by activating MMPs responsible for the degradation of skin collagen and inhibiting collagen synthesis of ECM in connective tissues (26). In this study, we observed the upregulation of MMPs (especially MMP-1) and the degradation of dermal collagen following UV irradiation. Porphyra-334 was a potent suppressor of UVA-induced MMP generation, and it also inhibited the MMP-1-initiated degradation of type I collagen. Therefore, inhibition of collagenase MMP expression or activation of collagen synthesis may be an effective strategy to prevent wrinkle formation following UVA irradiation. This mechanism predicts that a free radical scavenger may prevent UV-induced dermal damage by inhibiting MMP induction. These findings suggest that the regulation of MMPs and type I collagen levels in UV-irradiated fibroblasts may be related to the inhibition of ROS generation by porphyra-334. MAAs from red algae are known to effectively decrease MMPs and increase type I collagen expression levels to prevent premature skin aging (27). Results of this study suggest that porphyra-334 increases procollagen production by suppressing MMP gene expression and reducing MMP production, which can reduce cytokine secretion by human skin fibroblasts. Many algae have been screened for potential MMP inhibitors. For example, *Corallina pilulifera* (28), *Ecklonia cava* (29), *Laurencia undulata* (30) and *Amphiroa dilatata* (31) exhibited inhibitory effects on MMP activities. In this study, porphyra-334 was safe to human skin fibroblasts and significantly decreased the expression of MMPs induced by UVA irradiation.

Wrinkle formation in the skin is closely associated with the degradation of ECM, and UV irradiation is known to induce the degradation of ECM (32). UV irradiation enhances collagenase activity and contributes to wrinkle formation through the degradation of collagen in the dermal ECM (33,34). Therefore, collagenase inhibitors have been identified as potential therapeutic agents that protect against photoaging and wrinkle formation (35). Collagen is the main component of the ECM of dermal connective tissue, and its concentration decreases with photoaging. Once collagen is initially cleaved by MMP-1, MMP-13 and other MMPs, collagen breakdown is further promoted. The enzyme mainly responsible for collagen breakdown in skin is MMP-1, which cleaves types I, III, VII, VIII and X collagen. As reported previously with increasing photoaging, MMP-1 levels increase and collagen synthesis decreases in sun-protected human skin *in vivo* (36). In this study, the exposure of human skin fibroblasts to UVA significantly decreased type I collagen levels and increased MMP secretion, both of which were reversed by porphyra-334. Elastin is important in the dermis (37), and UV exposure has been shown to cause elastin degradation by activating elastase (38). As shown in Fig. 4B, the inhibitory effect of porphyra-334 on elastase activity was significant at a concentration of ≥10 μM.

Overall, porphyra-334 of *P. yezoensis* significantly inhibits ROS production, reduces MMP expression, and induces type I collagen, elastin at the mRNA and protein levels in a dose-dependent manner. These data suggest that porphyra-334 is a potential candidate for the prevention and treatment of skin photoaging. Additionally, porphyra-334 can be used to characterize the signal transduction pathways and molecular mechanisms involved in the anti-photoaging process.
